# A premature luteinizing hormone surge without elevated progesterone levels has no adverse effect on cumulative live birth rate in patient undergoing a flexible GnRH antagonist protocol: a retrospective study

**DOI:** 10.1186/s13048-023-01219-w

**Published:** 2023-06-27

**Authors:** Yangyang Zhang, Yang Xu, Jiao Yu, Xi Wang, Qing Xue, Jing Shang, Xiuli Yang, Xuemin Shan

**Affiliations:** Department of Obstetrics and Gynecology, Peking University First Hospital, 100034 Beijing, China

**Keywords:** Premature luteinizing hormone surge, Gonadotropin-releasing hormone antagonist, Cumulative pregnancy rate, Cumulative birth live rate, Pregnancy outcome

## Abstract

**Background:**

A premature luteinizing hormone (LH) surge refers to an endogenous LH peak that occurs before follicle maturation or human chorionic gonadotropin injection in the process of controlled ovarian hyperstimulation. The effect of premature LH surge on pregnancy outcomes in fresh embryo transfer cycles is still controversial. The aim of this study was to explore the effect of a premature LH surge without elevated progesterone levels on the cumulative pregnancy rate (CPR) and cumulative live birth rate (CLBR) of patients during a flexible GnRH antagonist protocol.

**Methods:**

A total of 730 infertile women undergoing IVF/ICSI were recruited for this retrospective study. Only women who either delivered a live infant or had no remaining frozen embryos after a single stimulation cycle were included in the analysis. During the study period, each patient underwent a flexible GnRH antagonist protocol. Women were divided into two groups according to the presence or absence of a premature LH surge. The primary outcome measures were the CPR and CLBR per ovarian stimulation cycle. The secondary outcome measures were the number of oocytes retrieved, fertilization rate, good-quality embryo rate, and clinical pregnancy rate.

**Results:**

Ninety-one women (12.47%) experienced a premature LH surge without elevated progesterone levels, and the other 639 (87.53%) women were assigned to the control group. The numbers of oocytes retrieved and fertilization rate were significantly greater in the premature LH surge group than in the control group. There was no significant difference between groups in the good-quality embryo rate, clinical pregnancy rate or live birth rate in the fresh embryo transfer cycle. The primary outcome measures, the CPR and CLBR per ovarian stimulation cycle, were not significantly different between the premature LH surge group and the control group. According to the analysis stratified by ovarian response (normal or high), there were no significant differences in pregnancy outcomes between the groups with and without a premature LH surge.

**Conclusions:**

The retrospective study demonstrated that the patients experiencing a transient premature LH surge without progesterone elevation had equivalent pregnancy outcomes with those without a premature LH surge on a flexible GnRH antagonist protocol. The present conclusions need to be further validated in a prospective well-designed large-scale study.

## Background

Luteinizing hormone (LH) is a glycoprotein gonadotropin synthesized and secreted by the basophils of the anterior pituitary gland. It can act together with follicle stimulating hormone (FSH) to promote follicle maturation, and induce ovulation and luteinization. LH is secreted in a pulsatile manner and is regulated by hypothalamic gonadotropin-releasing hormone (GnRH), ovarian estrogen, progesterone and inhibin. In the natural ovulatory menstrual cycle, LH plays an important role in normal folliculogenesis and oocyte maturation [1]. During the follicular phase, LH stimulates theca cells to synthesize androgens and provide substrates for estrogen synthesis. During the ovulation phase, the LH peak promotes oocyte maturation and induces ovulation. During the luteal phase, LH promotes progesterone and estrogen synthesis and maintains luteal function. Some researchers have proposed the concept of an “LH clinical treatment window”, in which LH levels lower than the threshold or higher than the “LH ceiling” negatively affect follicular development and endometrial receptivity [2].

In the process of controlled ovarian hyperstimulation(COH), gonadotropin (Gn) can promote the development of multiple follicles and increase estrogen levels, which may trigger positive feedback to induce LH release by the pituitary gland and thus evoke an endogenous LH peak. GnRH antagonists have been used to suppress pituitary activity, prevent premature LH surges and premature ovulation before follicular maturation during COH since the 1990s [3], although some women still experience this surge [4]. A premature LH surge refers to an endogenous LH peak that occurs before follicle maturation or human chorionic gonadotrophin (HCG) injection. At present, the criteria for a premature LH surge are vary and are controversial. A premature LH surge is defined as an LH level of 10 IU/L or higher with a progesterone level of 2 ng/ml or less [4, 5], but some studies have defined a premature LH surge as an LH level greater than threefold higher than that on day 2 of the same menstrual cycle [6]. The effect of premature LH surge on pregnancy outcomes in fresh embryo transfer cycles is still controversial. Some studies have demonstrated that a premature LH surge was associated with a decline in the clinical pregnancy rates [4, 7, 8], whereas others showed that a transient premature LH surge had no adverse effect on pregnancy rate [9]. The inconsistent findings may be due to differences in inclusion criteria, the definition of a premature LH surge and differences in protocols. Only one study has reported the effect of a premature LH surge on the cumulative live birth rate (CLBR). The result indicated that the premature LH rise was associated with decreased rates of cumulative live birth rate in patients of advanced age (≥ 37 years) [10]. The limitation of the above study was that it did not exclude the accompanying elevated serum progesterone level. As we all know, the premature LH surge may be followed by an elevated serum progesterone level. While the elevated progesterone level in the process of COH has been proved to affect endometrial receptivity and embryo quality and lead to a decrease in pregnancy rate. Therefore, a study could better assess the effect of a premature LH surge on CLBR only after excluding the accompanying elevated progesterone.

The aim of this study was conducted to explore the effect of a premature LH surge without elevated progesterone levels on the CLBR in patient undergoing a flexible GnRH antagonist protocol.

## Materials and method

### Patients

**Fig. 1:**
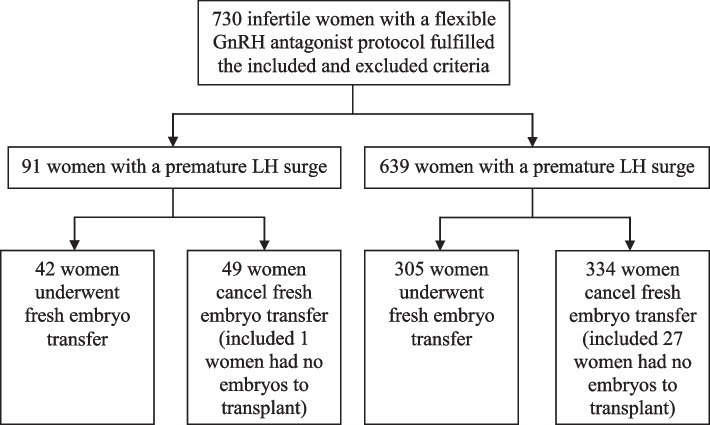
The flow diagram of participants recruited

In this retrospective study, a total of 730 infertile women undergoing in vitro fertilization (IVF)/intracytoplasmic sperm injection (ICSI) were recruited from January 2019 to December 2020 at the Reproductive and Genetic Medical Center of Peking University First Hospital (Fig. 1). Only women who either delivered a live infant or who had no remaining frozen embryos after a single aspiration cycle were included in the analysis. Patients were excluded if they fulfilled one of the following criteria: (1) age ≥ 40 years; (2) basal LH ≥ 10 IU/L; (3) poor ovarian response: number of oocyte retrieved ≤ 3; (4) progesterone > 2 ng/ml on the HCG day. In the study, a premature LH surge was defined as an LH level ≥ 10 IU/L with a progesterone level of 2 ng/ml or less. Women were divided into two groups according to the presence (Group A) or absence (Group B) of a premature LH surge. This study was approved by the Clinical Research Institutional Review Board of Peking University First Hospital (No. 2021–521).

### COH protocols

During the study period, each patient underwent a flexible GnRH antagonist protocol. The basal hormonal measured was performed on the 2nd or 3rd day of menstruation of stimulation cycle. A daily dose of 150–300 IU FSH was started on Day 2 or 3 of the menstrual cycle, and the dose was adjusted according to follicular development and hormone levels in the patients. A daily dose of 0.25 mg GnRH antagonist was initiated when the dominant follicle was ≥ 14 mm or a premature LH surge was recognized. Recombinant HCG was administered subcutaneously when the dominant follicle was 18–20 mm in diameter. Oocytes were collected by transvaginal ultrasound-guided follicular aspiration within approximately 36 h after the HCG trigger. Oocytes were fertilized by conventional IVF/ICSI, and embryos were transferred on Day 3 after oocyte retrieval. Luteal support was started on the day of oocyte retrieval. Fresh embryo transfer was cancelled for the following reasons: (1) to prevent the occurrence of ovarian hyperstimulation syndrome; (2) when no transferable embryos were obtained; (3) complicated with diseases unsuitable for fresh cycle transplantation, such as endometriosis or adenomyosis.

### Frozen embryo transfer

Embryos were cryopreserved if they met the following criteria: day 3 embryos with at least six blastomeres and ≤ 20% fragmentation or day 5–6 blastocysts at a minimum of expansion stage 3 with an inner cell mass score of A, B or C and a trophectoderm score of A, B or C. For frozen embryo transfer cycles, endometrium preparation protocols were determined by the patient’s menstrual cycle, including the natural cycle and the hormone replacement cycle. Progesterone preparation for luteal support was started 3 days before day-3-embryo transfer or 5 days before blastocyst transfer.

HCG tests were performed on day 14 after embryo transfer, and if the result was positive, luteal support was continued until 10 weeks of gestation. Clinical pregnancy was defined as the presence of an intrauterine gestational sac 4 weeks after embryo transfer. A live birth was defined as any birth event in which at least one baby was born alive after 28 weeks’ gestation.

### Main outcome measures

The primary outcome measures were the cumulative pregnancy rate (CPR) and CLBR per oocyte retrieval cycle. CPR was defined as pregnancy episodes in fresh and subsequent frozen-thawed embryo transfer cycles, and only the first pregnancy was included in the analysis. CLBR was defined as live birth episodes in fresh and subsequent frozen-thawed embryo transfer cycles, and only the first live birth was included in the analysis. The live birth of a singleton, twin, or other multiples is registered as one live birth [11]. The secondary outcome measures were the number of oocytes retrieved, fertilization rate, good-quality embryo rate, and clinical pregnancy rate.

The following criteria were used to define the ovarian response according to oocyte yield [12]: normal ovarian response, oocyte yield ≥ 4 and ≤ 15; and high ovarian response, oocyte yield > 15.

### Statistical analysis

All analyses were performed with Software Package for Social Sciences (SPSS) version 13.0 for Windows. All normally distributed measurement data are expressed as the mean ± standard deviation (SD) after analysis with independent sample t-tests. Categorical data are presented as the number of cases and corresponding percentage after analysis by the chi-square test. When there is cell with an expected value less than 5, Fisher's exact test is performed. When there are cells with an expected value less than 1, the chi-square test cannot be performed. *P* < 0.05 was considered to indicate statistical significance.

## Results

In this study, 91 women (12.47%) experienced a premature LH surge (LH range, 10.02-45.52 IU/L), and the other 639 (87.53%) women were included as controls. As shown in Table 1, there were no significant differences in age, body mass index (BMI), basal FSH, antral follicle count (AFC) or anti-Müllerian hormone (AMH) between the groups with and without a premature LH surge. Women with a premature LH surge had a higher basal LH level than those in the control group.

**Table 1:** Baseline characteristics between women with or without a premature LH surge

Parameters	Group A (*n* = 91)	Group B (*n* = 639)	*p* value
Age (years)	31.91 ± 3.22	32.67 ± 3.53	0.054
BMI (kg/m^2^)	23.00 ± 3.34	22.47 ± 3.14	0.138
Basal FSH (mIU/mL)	7.98 ± 2.19	8.33 ± 2.83	0.182
Basal LH (mIU/mL)	5.66 ± 1.91	4.38 ± 1.96	< 0.001*
No. of AFCs (n)	15.76 ± 7.00	14.33 ± 6.90	0.066
AMH level (ng/ml)	4.24 ± 2.28	3.90 ± 2.11	0.159

^*^Indicates a significant difference (*P* < 0.05)

During COH process, some hormone levels (including LH, E2 and progesterone levels on the initiation day of GnRH antagonist, E2 and progesterone levels on the HCG trigger day) were significantly higher, the numbers of oocytes retrieved and MII oocytes were significantly greater, the fertilization rate was significantly higher and endometrial thickness on the HCG trigger day was significantly thicker in the premature LH surge group than in the control group. No differences between groups were found in the total dose of Gn, LH level on the HCG trigger day, or good-quality embryo rate (Table 2). On the first day after GnRH antagonist treatment was initiated, the LH level in all patients with a premature LH surge dropped below 10 IU/L.

**Table 2:** Comparison of treatment-related characteristics during COH between women with or without a premature LH surge

Parameters	Group A (*n* = 91)	Group B (*n* = 639)	*p* value
LH on the initiation day of GnRH antagonist (mIU/mL)	15.83 ± 6.95	4.63 ± 2.38	< 0.001*
E2 on the initiation day of GnRH antagonist (pg/ml)	2009.44 ± 979.88	1555.32 ± 1143.41	0.001
Progesterone on the initiation day of GnRH antagonist (pg/ml)	1.14 ± 0.52	0.81 ± 0.42	< 0.001*
Total Gn dose (IU)	2362.25 ± 913.92	2534.29 ± 911.04	0.098
Endometrial thickness on HCG day (mm)	11.08 ± 1.87	10.51 ± 2.20	0.019*
LH on HCG day (mIU/mL)	2.80 ± 2.47	2.38 ± 1.77	0.125
E2 on HCG day (pg/ml)	3996.03 ± 1866.51	3205.08 ± 1637.49	< 0.001*
Progesterone on HCG day (pg/ml)	1.13 ± 0.43	1.04 ± 0.40	0.049
Oocytes retrieved	12.18 ± 6.06	10.57 ± 5.56	0.011*
MII oocytes	8.77 ± 6.57	7.40 ± 5.69	0.036*
Fertilization rate	75.81% (840/1108)	72.51% (4898/6755)	0.022*
Good-quality embryo rate	36.15% (282/780)	39.81% (1815/4559)	0.053
Transfer cancellation rate	53.85% (49/91)	52.27% (334/639)	0.778

^*^Indicates a significant difference (*P* < 0.05)

Among the 730 infertility patients, 347 women underwent fresh embryo transfer. The embryo transfer cancellation rates were 53.85% for women with a premature LH surge and 52.27% for women without premature LH surge. There was no significant difference in the clinical pregnancy rate or live birth rate in the fresh embryo transfer cycle between the groups with and without a premature LH surge. In this study, 28 women had no embryos to transplant, and there was one woman had premature LH surge. Among the 730 infertility patients, the primary outcome measures, the CPR and CLBR per ovarian stimulation cycle, were not significantly different between the premature LH surge group and the control group (Table 3).

**Table 3:** Comparison of pregnancy outcomes between women with or without a premature LH surge

Parameters	Group A (*n* = 91)	Group B (*n* = 639)	*p* value
No. of embryos transferred per ET (n)	1.88 ± 0.33	1.92 ± 0.28	0.424
Clinical pregnancy rate	57.14% (24/42)	49.51% (151/305)	0.354
Live birth rate	45.24% (19/42)	41.64% (127/305)	0.658
Cumulative pregnancy rate	73.63% (67/91)	67.14% (429/639)	0.215
Cumulative live birth rate	67.03% (61/91)	59.78% (382/639)	0.185

Among the 730 infertility patients, 593 had a normal ovarian response, and 137 had a high ovarian response. A premature LH surge occurred in 68 women (11.47%) in the normal ovarian response group, and 23 women (16.79%) in the high ovarian response group. According to the analysis stratified by ovarian response, whether in normal ovarian response or high ovarian response, there were no significant differences in pregnancy outcomes between women with and without a premature LH surge (Table 4).

**Table 4:** Comparison of pregnancy outcomes between women with or without a premature LH surge based on ovarian response

	Normal ovarian response (*n* = 593)			High ovarian response (*n* = 137)		
	Group A (*n* = 68)	Group B (*n* = 525)	*P* value	Group A (*n* = 23)	Group B (*n* = 114)	*P* value
Clinical pregnancy rate	60.00% (24/40)	48.99% (145/296)	0.191	0 (0/2)	66.67% (6/9)	-
Live birth rate	47.50% (19/40)	41.55% (123/296)	0.475	0 (0/2)	44.44% (4/9)	-
Cumulative pregnancy rate	64.71% (44/68)	62.86% (330/525)	0.766	100% (23/23)	86.84% (99/114)	-
Cumulative live birth rate	57.35% (39/68)	55.62% (292/525)	0.786	95.65% (22/23)	78.95% (90/114)	0.075

## Discussion

In a fixed GnRH antagonist protocol, a GnRH antagonist is initiated on Day 5-6 of COH, while in a flexible GnRH antagonist protocol, a patient starts receiving an antagonist when the dominant follicle reaches ≥14mm in size. Studies have shown that GnRH antagonists can inhibit endogenous LH peaks and avoid premature ovulation induced by LH peaks before follicular maturation. However, some women still experience a premature LH surge before GnRH antagonist administration. Researchers have identified transient premature LH surges was found in women on both the fixed and flexible GnRH antagonist protocols [7, 8]. However, studies have shown that patients on the flexible protocol were more prone to a premature LH surge than those on the fixed protocol [13]. Transient LH suppression by a GnRH antagonist is achieved by competitive inhibition of the GnRH receptor, but endogenous estrogen-induced GnRH release can still occur; thus, in a small proportion of patients, antagonist cycles fail to control the LH surge [14, 15].

Our study showed that the incidence of a premature LH surge without elevated progesterone levels on the flexible GnRH antagonist protocol was 12.47%. Compared with previous studies [7, 8], this study found a lower premature LH surge rate, which may be related to the different definitions of a premature LH surge and the different criteria. For example, in the study of Zhang et al., a premature LH surges was defined as either more than threefold of the basic LH level on day 2 of the same menstrual cycle; or the absolute value > 10IU/L [8]. The underlying mechanisms of a premature LH surge are poorly identified, but are potentially related to a positive feedback loop between high E2 concentrations and the pituitary gland during ovulation stimulation [16]. Our study showed that the numbers of oocytes retrieved and the fertilization rate were higher in the premature LH surge group, meanwhile, the good-quality embryo rate were comparable among the women with and without a premature LH surge, which suggested that a premature LH surge did not affect the development and quality of oocyte. In general, elevated LH levels are accompanied by elevated progesterone levels, which can lead to premature transformation of the endometrium and discordance between embryo development and the endometrium, resulting in a low pregnancy success rate after fresh cycle transfer. In addition, it has been suggested that a transient premature LH surge without a progesterone elevation during COH can also lead to a reduced clinical pregnancy rate [7, 8]. Geng et al. reported that women with a premature LH rise had significantly poorer pregnancy outcomes than those without such a rise among ovarian high responders undergoing the GnRH antagonist stimulation protocol. An AFC of 22 or higher and an E2 level of 669 pg/mL or higher on the day of GnRH antagonist administration were predictive factors of a premature LH rise [7]. In a retrospective study of 405 women undergoing a fixed GnRH antagonist protocol, the results showed that a transient premature LH surge without elevated serum progesterone was associated with poor pregnancy outcomes in fresh embryo transfer cycles [8]. In contrast, our study showed that there was no decrease in the clinical pregnancy rate or live birth rate in the fresh transfer cycle among women with a premature LH surge. These results are consistent with Kummer et al. findings, which demonstrated that a transient LH rise was not associated with a decline in fertilization, implantation, or pregnancy rate per embryo transfer [9].

Meanwhile, the main finding of our study was that the CPR and CLBR were comparable among the women with and without a premature LH surge, with 67.03% and 59.78% of participants, respectively, achieving a live birth. According to the analysis stratified by ovarian response, a transient LH rise was not associated with a decline in pregnancy outcomes. Gao et al. reported that the premature LH rise was associated with decreased rates of cumulative live birth rate in patients of advanced age (≥37 years) [10]. The inconsistent finding may be due to that they did not exclude the accompanying elevated serum progesterone level. Therefore, we concluded that a transient premature LH surge without progesterone elevation during COH had no adverse effect on oocyte development. This result depends on the GnRH antagonist protocol and the antagonist itself, which can quickly and effectively decrease endogenous LH levels with a limited effect on endogenous FSH. At present, the most widely used GnRH antagonists are Cetrorelix and Ganirelix. Studies have shown that both GnRH antagonists effectively decrease LH levels with no significant differences in pregnancy outcomes [17, 18]. In our study, on the first day after initiating GnRH antagonist treatment, the LH level in all patients with a premature LH surge dropped below 10 IU/L. Therefore, with the flexible antagonist protocol, a transient premature LH surge had no adverse effect on pregnancy outcome if a GnRH antagonist was administered immediately after a premature LH surge.

Our study found that the E2 levels on the HCG trigger day, numbers of oocytes retrieved and MII oocytes were higher in the premature LH surge group than in the control group. In addition, a stratified subgroup analysis indicated that women with a high ovarian response were more prone to a premature LH surge than those with a normal ovarian response. The incidence of a premature LH surge in patients with a high ovarian response was higher than in those with a normal ovarian response, and this finding is consistent with previous findings [4]. The underlying mechanisms are incompletely understood, but it may be that compared with patients with normal ovarian response, patients with a high ovarian response have more follicles and higher estrogen levels, which increases the chance that pituitary positive feedback is induced, thus increasing the likelihood of experiencing a premature LH surge [16].

The limitation of our study is that it is a retrospective study with a relatively small sample size, which prevents statistical detection of further clinically significant differences. Therefore, the present conclusions need to be further validated in a prospective well-designed large-scale study. In addition, the definition and criteria for a premature LH surge are not consistent at present, and further investigation will be performed according to the different definitions.

## Conclusions

The retrospective study demonstrated that the patients experiencing a transient premature LH surge without progesterone elevation had equivalent CLBR with those without a premature LH surge on a flexible GnRH antagonist protocol. In conclusion, a premature LH surge does not impair the pregnancy outcomes if a GnRH antagonist was administered as soon as a premature LH surge occurred and therefore should not be a reason to cancel to cycle.

## Data Availability

The datasets used and/or analyzed during the current study are available from the corresponding author on reasonable request.

## Abbreviations

AMH: Anti-Müllerian hormone
BMI: Body mass index
CLBR: Cumulative live birth rate
COH: Controlled ovarian hyperstimulation
CPR: Cumulative pregnancy rate
E2: Estradiol
FSH: Follicle stimulating hormone
Gn: Gonadotropin
GnRH: Gonadotropin-releasing hormone
HCG: Human chorionic gonadotrophin
ICSI: Intracytoplasmic sperm injection
IVF: In vitro fertilization
LH: Luteinizing hormone
